# Supplementary material for: A network dynamical simulation model for the study of antibiotic resistance in microbial communities
Source: Gut Microbes. 2026 Sep 19;18(1):2734703. doi: 10.1080/19490976.2026.2734703 (PMC13596919; doi:10.1080/19490976.2026.2734703)
Supplement: Supplementary file 1 [file KGMI_A_2734703_SM9053.zip › supplementary_file_1/Figures/er_delta_gc_size.pdf]

Erd.s−Rényi networks: mean change in giant component size

Numbers inside tiles show mean ± SD across simulation replicates

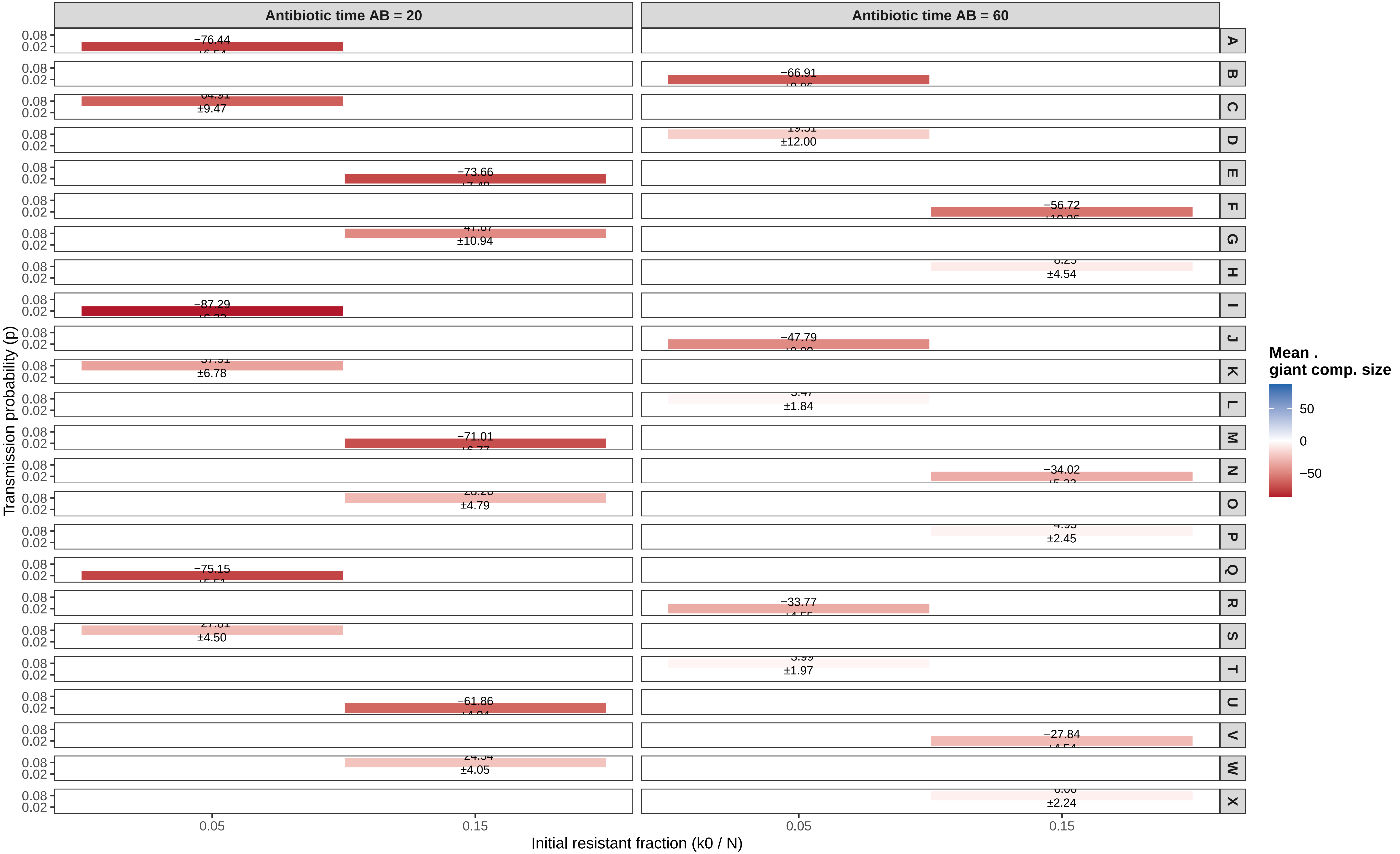

Panel key: A = p_edge 0.02 | B = p_edge 0.02 | C = p_edge 0.02 | D = p_edge 0.02 | E = p_edge 0.02 | F = p_edge 0.02 | G = p_edge 0.02 | H = p_edge 0.02 | I = p_edge 0.05 | J = p_edge 0.05 | K = p_edge 0
